# Transcriptional response of Mexican axolotls to *Ambystoma tigrinum *virus (ATV) infection

**DOI:** 10.1186/1471-2164-9-493

**Published:** 2008-10-20

**Authors:** Jennifer D Cotter, Andrew Storfer, Robert B Page, Christopher K Beachy, S Randal Voss

**Affiliations:** 1 School of Biological Sciences, Washington State University, Pullman, WA 99164-4236, USA; 2 Department of Biology and Spinal Cord and Brain Injury Research Center, University of Kentucky, Lexington KY 40506, USA; 3 Department of Biology, Minot State University, Minot ND 58707, USA

## Abstract

**Background:**

Very little is known about the immunological responses of amphibians to pathogens that are causing global population declines. We used a custom microarray gene chip to characterize gene expression responses of axolotls (*Ambystoma mexicanum*) to an emerging viral pathogen, *Ambystoma tigrinum *virus (ATV).

**Result:**

At 0, 24, 72, and 144 hours post-infection, spleen and lung samples were removed for estimation of host mRNA abundance and viral load. A total of 158 up-regulated and 105 down-regulated genes were identified across all time points using statistical and fold level criteria. The presumptive functions of these genes suggest a robust innate immune and antiviral gene expression response is initiated by *A. mexicanum *as early as 24 hours after ATV infection. At 24 hours, we observed transcript abundance changes for genes that are associated with phagocytosis and cytokine signaling, complement, and other general immune and defense responses. By 144 hours, we observed gene expression changes indicating host-mediated cell death, inflammation, and cytotoxicity.

**Conclusion:**

Although *A. mexicanum *appears to mount a robust innate immune response, we did not observe gene expression changes indicative of lymphocyte proliferation in the spleen, which is associated with clearance of Frog 3 iridovirus in adult *Xenopus*. We speculate that ATV may be especially lethal to *A. mexicanum *and related tiger salamanders because they lack proliferative lymphocyte responses that are needed to clear highly virulent iridoviruses. Genes identified from this study provide important new resources to investigate ATV disease pathology and host-pathogen dynamics in natural populations.

## Background

Emerging infectious diseases (EIDs) pose a serious threat to the health, stability, and persistence of human and wildlife populations [1-4]. Genetic and genomic tools have been incredibly useful for discovery of genes associated with host response and variation in resistance or susceptibility to a variety of pathogens [5-7]. The advent of genomic tools such as microarray analysis has offered new insights into host-pathogen systems. Additionally, their application to genomic response to host disease response allows rapid characterization of candidate genes for further research into control and eradication methods.

EIDs are a leading hypothesis for the global decline of amphibians and two pathogens in particular, *Batrachochytrium dendrobatidis *and Ranaviruses have been implicated in worldwide epizootics. Although studies are beginning to investigate possible mechanisms of resistance to these pathogens [8], in general, very little is known about the immune response of amphibians to EIDs. This is because most natural amphibian species are not used as laboratory models and we lack fundamental molecular tools to investigate disease pathology and host-pathogen interactions at the molecular level for all but a few species (e.g., *Ambystoma tigrinum spp., Xenopus spp*.).

Over the last 15 years, *Ranavirus *infections have been associated with marked increases in morbidity and mortality in fish, reptiles, and amphibians [9]. Ranaviruses are globally-distributed double-stranded, methylated DNA viruses of fish, amphibians and reptiles and are implicated in amphibian epizootics worldwide [9-11]. Both encapsulated and non-encapsulated forms can be infectious. The virus enters the cell via receptor mediated endocytosis or via fusion with the plasma membrane; and DNA and RNA synthesis occur in the nucleus, while protein synthesis occurs at morphologically specific assembly sites in the cytoplasm [9]. In North America, ranaviruses have been isolated from the majority of recent documented amphibian epizootics [12], including from tiger salamander (Ambystoma tigrinum) epizootics in Saskatchewan, Canada [13], Arizona [14], North Dakota, Utah, and Colorado, USA [15,16]. The viral variant that infects tiger salamanders, ATV, is transmitted either via direct contact with an infected animal or immersion in water that contains virus and infected individuals exhibit systemic hemorrhaging, edema, ulceration, and necrosis of the integument and internal organs [13,17,18]. In cases where ATV infection leads to mortality, it usually occurs within 2–3 weeks of exposure, with animals displaying symptoms often between 8–10 days post-exposure. Thus, ATV can rapidly overwhelm the tiger salamander immune response. However, mortality is not always a pathological endpoint because virulence and resistance are known to vary among ATV strains and tiger salamander populations, respectively, as indicated by both laboratory experiments and field observations [19]. Research characterizing the tiger salamander genomic response to ATV is needed to better understand the pathology, virulence, and possible mechanisms of resistance to this emerging disease.

The tiger salamander species complex includes *A. mexicanum *(Mexican axolotl), a model organism with a growing genomic and informatics resource base [20]. The immune system of the Mexican axolotl has been extensively studied using several classical approaches. Relative to other vertebrate models, the axolotl immune response has been described as immunodeficient [21,22]. There are several reasons for this characterization, including: production of only two immunoglobulin (Ig) classes, only one of which regulates the humoral response and neither of which is anamnestic [23,24]; no response to soluble antigens [25]; poor mixed lymphocyte reactions [26,27]; and lack of cellular cooperation during the humoral immune response as indicated by enhanced humoral immunity following thymectomy or X-ray irradiation [28,29]. Weak immune responses are known for salamanders in general, and the Mexican axolotl and related tiger salamanders are especially susceptible to ATV infections with high observed mortality rates both in the laboratory and in the field. Indeed, an outbreak of ATV in 2003 at the Indiana Axolotl Colony significantly reduced adult stocks before the virus was contained. By way of comparison, adult *Xenopus *effectively clear close-related FV3 *Ranavirus *with an immune response that includes an early T-cell proliferative phase in the spleen [30].

To further investigate the axolotl immune response to ATV, we used an Affymetrix custom microarray to identify genes that were significantly, differentially expressed in the spleen. We then compared these genes to a list of genes associated with regeneration that were previously identified from *A. mexicanum *using the same microarray platform. We reasoned that such a comparison would allow us to filter gene expression responses of humoral cells induced generally in response to injury and stress from those expressed specifically in response to ATV infection. Also, this comparison would potentially identify gene expression signatures associated with cell proliferation in response to ATV, as we have previously identified many cell proliferation probe sets on the *Ambystoma *genechip that are differentially regulated during spinal cord regeneration [31]. The genes that we describe provide mechanistic insights and new tools to investigate salamander antiviral responses in the laboratory and in natural populations.

## Methods

### Animal care and surgery protocols

Inbred *A. mexicanum *eggs from a single full-sib mating were obtained from the Ambystoma Genetic Stock Center at the University of Kentucky. Each *A. mexicanum *egg and larva was reared in an individual container in aquifer water treated with ReptiSafe and changed weekly. Individuals were fed brine shrimp *ad libitum *for the first four weeks post-hatching and blackworms (Tubifex) *ad libitum *thereafter. Animals were reared in an environmental chamber on a 12:12 h light:dark cycle at 20°C. At 4.5 months of age, 12 individuals were injected with 100 μl of 10^6 ^p.f.u./ml of ATV isolated from the axolotl colony and suspended in cell culture medium. This amount of virus was determined to be the minimum lethal dose via injection in previous unpublished experiments (Storfer, unpublished data) and the strain utilized in the experiment was extracted from axolotls that had previously been infected and killed by the virus. Simultaneously, four uninfected (control) individuals were sacrificed in MS222 for spleen and lung removal. Spleens from all animals were flash frozen in liquid nitrogen. The same surgical procedure was performed on four infected individuals following 24, 72 and 144 hours of infection. Spleen tissue was utilized due to its previously noted importance in CD8+ T cell immune responses to Ranaviruses, particularly FV3, in frogs [30] Additionally, spleen is an important immune organ as antigens from the blood are processed in the spleen. Lung tissue was removed for viral quantification as it is an internal organ that can be utilized in early stage virus quantification (Stewart, unpublished data).

During the infection period behavioral observations were taken opportunistically. Total RNA was extracted from spleen with TRIzol (Invitrogen) according to the manufacturer's protocol. RNA isolations were further purified using RNeasy mini columns (Qiagen). The amount of RNA present in each isolate was determined via UV spectrophotometry, and RNA quality was inspected via a 2100 Agilent Bioanalyzer. Sixteen high quality isolates (four replicates at each of four sampling times: 0 (controls), 24, 72, and 144 hours post-infection) were used to make individual-specific pools of biotin labeled cRNA probes. Each of the 16 pools was then independently hybridized to an Amby_001 custom Affymetrix GeneChip (for a more detailed description of the microarray platform see [31] and [32]). The University of Kentucky Microarray Core Facility generated cRNA probes and performed hybridizations according to standard Affymetrix protocols.

### Quality Control and Data Processing

All quality control and processing analyses were done in R [33]. We used the Bioconductor package "affy"  to perform several quality control analyses at the individual probe level [34,35]. These analyses included: (1) viewing images of the log(intensity) values of the probes on each GeneChip to check for spatial artifacts, (2) investigating measures of central tendency and dispersion by viewing box-plots and histograms of all the GeneChips, (3) viewing pair-wise M versus A plot matrices for replicate GeneChips, and (4) viewing an RNA degradation plot [35] that enables the visualization of the 3' labeling bias associated with all GeneChips simultaneously. Upon conducting these probe level analyses, we background corrected, normalized, and summarized all sixteen GeneChips using the Robust Multi-array Average (RMA) algorithm [36]. Following this, we calculated correlation matrices for replicate GeneChips (four correlation matrices with four GeneChips per matrix; all *r *from replicate GeneChips > 0.980) on the summarized probe-set level data. The strong correlations observed between replicate GeneChips suggests that we were able to obtain a high degree of repeatability within treatments.

### Data Filtering

Microarrays may not accurately quantify the abundance of lowly expressed genes [37]. Calculating statistical tests for such genes adds to the multiple testing burden that is inherent to microarray studies. To address this issue, we filtered genes whose mean intensity across all 16 GeneChips was greater than the mean of the lowest quartiles (25^th ^percentiles) across all GeneChips (*n *= 16, mean = 5.83, SD = 0.06; data presented on a log_2 _scale). Upon imposing this filtering criterion, 3619 probe-sets were available for significance testing.

### Identifying Differentially Expressed Genes

We used the Bioconductor package LIMMA [38,39] to generate moderated *t*-statistics for all six of the possible pair-wise contrasts of the four sampling times investigated in our study. LIMMA employs an empirical Bayes methodology that effectively shrinks the sample variances towards a pooled estimate. This approach reduces the likelihood of obtaining large test statistics due to underestimation of the sample variances. The moderated *t*-statistics generated by LIMMA test the null hypothesis that the difference between the two groups being compared is zero (*i.e*., group 1 – group 2 = 0). LIMMA also generates moderated *F*-statistics that test the null hypothesis that none of the contrasts within a family of contrasts are statistically significant. We corrected for multiple testing by applying the step-up algorithm [40] to the *P*-values of the moderated *F*-statistics associated with our six contrasts. Upon correcting for multiple testing, we identified 2322 genes (probe-sets) that were statistically significant. To prioritize amongst differentially expressed genes, we focused on probe-sets that exhibited two-fold or greater changes at any time-point relative to controls. Any gene that was non-significantly down-regulated but significantly up-regulated at one or more time points was considered up-regulated, and vice versa for classification of up- versus down-regulation. We also required that these probe sets have moderated *F*-statistics greater than or equal to the 50^th ^percentile of the 2322 *F*-statistics from the statistically significant probe-sets (*F *≥ 12.68). We further limited our analysis to only those probe sets that exhibited significant sequence identity with a human reference sequence. We note that 263 probe-sets with no functional annotation were statistically significant, differentially expressed by ≥ two-fold, and had *F*-values ≥ 12.68.

### Clustering

Hybridization intensities were averaged within treatment groups (0, 24, 72, and 144 hrs post-infection) and log_2 _ratios were calculated for each non-zero sampling time relative to 0 hours post-infection. Genesis v. 1.6.0 [41,42] was used cluster these log_2 _ratio data and to generate heat maps. Clustering was conducted using a Self Organizing Map (SOM) algorithm. Default conditions were used with the exception that the SOM was allowed to run for 263,000 iterations. The dimensions of the final SOM are 2_x _*1_y_. These dimensions were determined by comparing output from several different combinations.

### Enrichment Analyses

Functional annotation of genes by gene ontology was performed using the Database for Annotation, Visualization and Integrated Discovery (DAVID, [43]). Functional annotation clustering was performed using the default settings with the exception of using the highest classification stringency.

### Quantitative real-time PCR

We used quantitative real-time PCR (qPCR) to confirm the results of the microarrays. We estimated a fold change for 24 and 72 hr time points using the ΔΔct method of relative quantification [44], utilizing ribosomal protein L 19 as an endogenous control gene. The same total RNA that was used for microarray analysis was used to create cDNA for qPCR using the BioRad iScript cDNA synthesis kit, following manufacturer instruction. Primers for the qPCR were designed using Primer Express 2.0 (Applied Biosystems). Primers were designed to encompass the sequence of GeneChip probe sets (Additional file 1). qPCR was accomplished using SYBR Green chemistry.

To verify that exposed animals were infected and to quantify viral load and replication over time, we performed qPCR on lung tissue with TaqMan chemistry following the protocol detailed in [45]. ANOVA with a Tukey's HSD correction for all pairwise comparisons was performed to determine if viral loads were significantly different across time points.

## Results

### Viral load and disease pathogenesis

**Figure 1:**
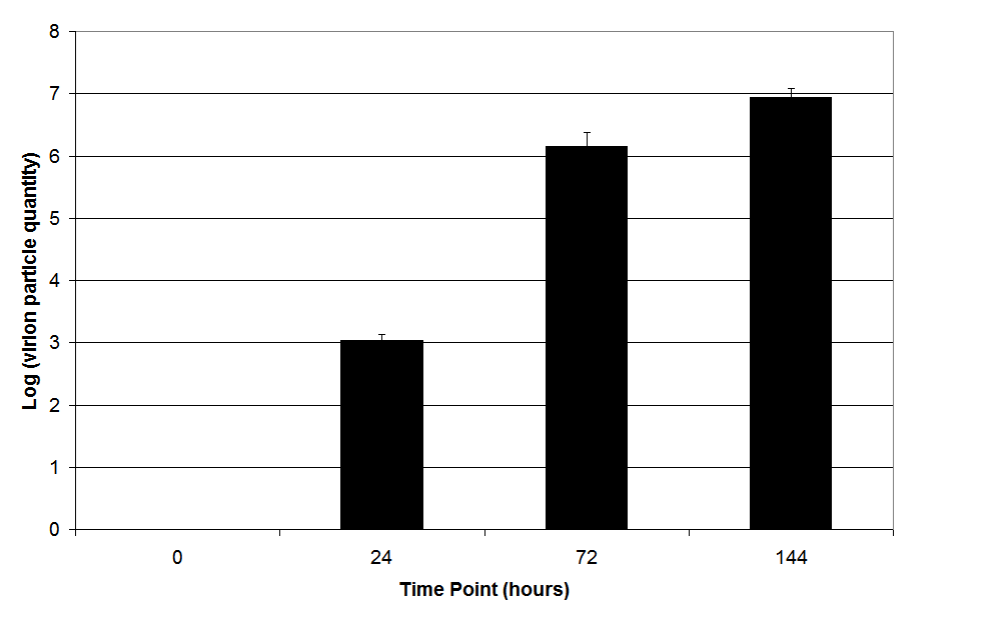
Log values of viral particles quantified with quantitative real-time PCR across all time points.

Viral load for each animal was estimated using qPCR and then averaged for each time point (Fig 1). The significant increase in viral load across time points indicates that animals were infected and that viral replication was occurring. ANOVA with a Tukey's correction for multiple comparisons confirmed that viral load increased linearly between 24, 72, and 144 hours post-infection, and all time points were significantly different from all other time points (F_3,44 _= 242.56; p ≤ 0.01).

No animals displayed any gross symptoms of ATV infection in terms of hemorrhaging, lesions or edema, either externally or on any internal organs upon euthanasia and subsequent surgery. Similarly, there were no notable changes in behavior observed during the period of infection. This is likely due to the relatively short infection period utilized in this experiment. As noted in the introduction, infected animals often take 8–10 days, or more, to become symptomatic upon infection.

### Gene clustering and functional annotation

**Figure 2:**
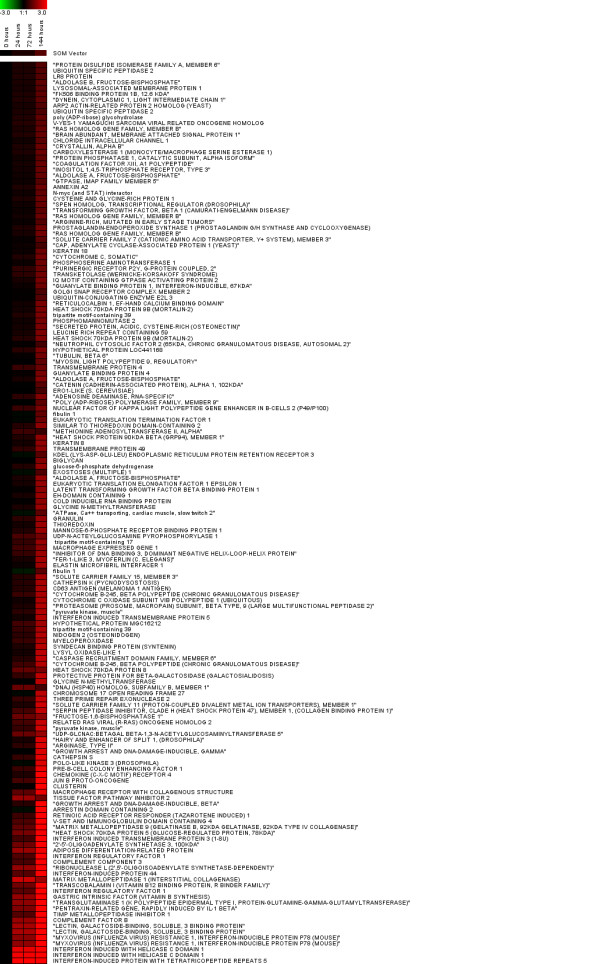
**Expression profiles for cluster 1.** Each row represents an individual gene, and each column a post-infection time point. Red coloration indicates increased expression of a gene relative to uninfected animals, and green indicates decreased expression. Genes (Cluster 1, n = 158) identified to be significantly up-regulated in response to ATV infection.

**Figure 3:**
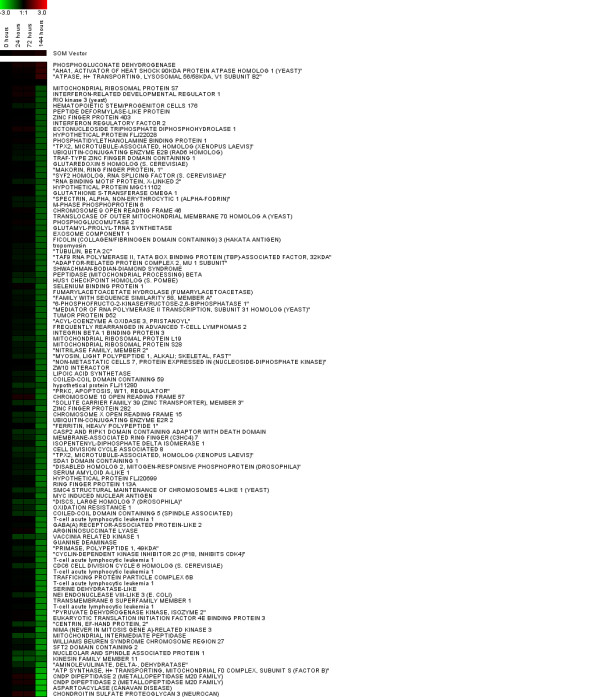
**Expression profiles for cluster 2.** Each row represents an individual gene, and each column a post-infection time point. Red coloration indicates increased expression of a gene relative to uninfected animals, and green indicates decreased expression. Genes (Cluster 2, n = 105) identified to be significantly down-regulated in response to ATV infection.

We identified 263 probe sets with statistically significant differences in mRNA abundances between Day 0 and any other subsequent time point (Tables 1, 2). We assume that statistically significant probe sets correspond to genes that were differentially regulated after ATV infection. Cluster analysis of the statistically significant genes identified two groups that exhibited similar changes in mRNA abundance. After ATV infection at Day 0, 158 putative genes showed a significant increase in mRNA abundance at subsequent time points (Figure 2), while 105 transcripts showed a significant decrease (Figure 3). Thus, more genes were up-regulated than down-regulated in response to ATV infection. Overall, DAVID categorized statistically significant genes among 44 different groups that correspond to different biological processes. Eight of these groups contained more genes than would be expected by chance sampling of genes from the microarray (geometric mean p-value < 0.05); these groups were considered significantly enriched with candidate genes relative to other groups (Table 3). Four of these significant groups contain gene ontologies related to immune response and pathogen response, including innate immunity, complement activation, lysosome function, and antigen processing and presentation. The most enriched functional group contains genes primarily related to immune function and defense responses. The remaining four functional groups contain gene ontologies related to ion binding, ion transport, vitamin metabolism, and response to an unfolded protein. Many genes that were classified in broader biological process categories that are not directly immunity-related are nonetheless associated with immunity in vertebrates [e.g. [46-48]].

**Table 1:** Genes which were significantly up-regulated at any time point. Numbers indicate fold change at that time point.

Gene ID	Gene Name	24 hr	72 hr	144 hr
immune response				
SRV_03329_at	INTERFERON-INDUCED PROTEIN WITH TETRATRICOPEPTIDE REPEATS 5	11.85	82.22	91.04
SRV_01342_at	INTERFERON REGULATORY FACTOR 1	1.94	12.71	12.79
SRV_01343_a_at	INTERFERON REGULATORY FACTOR 1	2.10	16.07	15.63
SRV_13637_a_at	INTERFERON INDUCED TRANSMEMBRANE PROTEIN 3 (1–8 U)	1.75	8.48	10.88
SRV_01199_a_at	CLUSTERIN	1.05	4.35	8.02
SRV_00442_at	SOLUTE CARRIER FAMILY 11 (PROTON-COUPLED DIVALENT METAL ION TRANSPORTERS), MEMBER 1	1.28	6.17	5.38
SRV_01303_a_at	GUANYLATE BINDING PROTEIN 1, INTERFERON-INDUCIBLE, 67 KDA	1.10	2.35	2.49
SRV_02828_at	2'-5'-OLIGOADENYLATE SYNTHETASE 3, 100 KDA	2.47	13.56	9.35
SRV_02072_at	CATHEPSIN S	1.54	5.51	6.43
SRV_02588_a_at	LECTIN, GALACTOSIDE-BINDING, SOLUBLE, 3 BINDING PROTEIN	4.04	26.37	24.40
SRV_02586_at	LECTIN, GALACTOSIDE-BINDING, SOLUBLE, 3 BINDING PROTEIN	3.88	21.30	18.03
				
response to virus				
SRV_03073_at	INTERFERON-INDUCED PROTEIN 44	2.05	9.44	13.16
SRV_01439_a_at	MYXOVIRUS (INFLUENZA VIRUS) RESISTANCE 1, INTERFERON-INDUCIBLE PROTEIN P78 (MOUSE)	3.13	29.23	36.80
SRV_01441_at	MYXOVIRUS (INFLUENZA VIRUS) RESISTANCE 1, INTERFERON-INDUCIBLE PROTEIN P78 (MOUSE)	3.26	28.24	35.84
SRV_04604_s_at	INTERFERON INDUCED WITH HELICASE C DOMAIN 1	6.81	27.34	24.15
SRV_04604_at	INTERFERON INDUCED WITH HELICASE C DOMAIN 1	7.27	25.43	22.62
SRV_04518_at	RIBONUCLEASE L (2',5'-OLIGOISOADENYLATE SYNTHETASE-DEPENDENT)	3.30	7.38	8.66
				
cell defense response				
SRV_00353_at	NEUTROPHIL CYTOSOLIC FACTOR 2 (65 KDA, CHRONIC GRANULOMATOUS DISEASE, AUTOSOMAL 2)	1.05	2.07	1.73
SRV_00264_a_at	MYELOPEROXIDASE	1.60	7.60	4.10
				
antigen processing and presentation				
SRV_05347_at	PROTEASOME (PROSOME, MACROPAIN) SUBUNIT, BETA TYPE, 9 (LARGE MULTIFUNCTIONAL PEPTIDASE 2)	1.27	3.73	4.25
				
lysosome/endosome				
SRV_02581_a_at	LYSOSOMAL-ASSOCIATED MEMBRANE PROTEIN 1	1.23	2.26	2.06
SRV_12596_at	EH-DOMAIN CONTAINING 1	1.22	4.16	3.47
				
inflammatory response				
SRV_10702_at	CYTOCHROME B-245, BETA POLYPEPTIDE (CHRONIC GRANULOMATOUS DISEASE)	1.69	5.58	4.40
SRV_00330_at	CYTOCHROME B-245, BETA POLYPEPTIDE (CHRONIC GRANULOMATOUS DISEASE)	1.59	4.74	3.89
SRV_01877_at	CHEMOKINE (C-X-C MOTIF) RECEPTOR 4	1.29	5.70	8.08
SRV_02292_at	N-myc (and STAT) interactor	1.15	2.29	2.29
SRV_00453_a_at	TRANSFORMING GROWTH FACTOR, BETA 1 (CAMURATI-ENGELMANN DISEASE)	1.12	2.19	2.18
SRV_01617_a_at	PENTRAXIN-RELATED GENE, RAPIDLY INDUCED BY IL-1 BETA	2.83	12.82	17.76
				
cell cycle				
SRV_02067_at	POLO-LIKE KINASE 3 (DROSOPHILA)	0.93	3.82	6.18
SRV_11882_s_at	RAS HOMOLOG GENE FAMILY, MEMBER B	1.18	2.33	2.40
SRV_02051_at	RAS HOMOLOG GENE FAMILY, MEMBER B	1.26	2.15	2.28
SRV_02050_at	RAS HOMOLOG GENE FAMILY, MEMBER B	1.23	2.51	2.55
SRV_00154_at	EXOSTOSES (MULTIPLE) 1	0.90	2.07	1.90
				
glycolysis				
SRV_01520_at	pyruvate kinase, muscle	1.41	4.60	4.22
SRV_01519_a_at	pyruvate kinase, muscle	1.56	5.94	5.48
B_s_at	ALDOLASE A, FRUCTOSE-BISPHOSPHATE	1.22	3.14	2.96
				
transcription				
SRV_01351_at	JUN B PROTO-ONCOGENE	1.76	7.82	7.54
SRV_01336_a_at	INHIBITOR OF DNA BINDING 3, DOMINANT NEGATIVE HELIX-LOOP-HELIX PROTEIN	1.56	3.14	3.57
SRV_02310_at	EUKARYOTIC TRANSLATION TERMINATION FACTOR 1	0.95	2.70	2.45
SRV_03646_at	SPEN HOMOLOG, TRANSCRIPTIONAL REGULATOR (DROSOPHILA)	1.15	2.22	2.30
SRV_02571_at	HAIRY AND ENHANCER OF SPLIT 1, (DROSOPHILA)	1.23	4.52	6.10
				
complement				
SRV_00137_a_at	COMPLEMENT COMPONENT 3	1.80	9.79	13.66
SRV_01145_a_at	COMPLEMENT FACTOR B	3.46	18.33	20.05
				
apoptosis				
SRV_02399_a_at	MATRIX METALLOPEPTIDASE 9 (GELATINASE B, 92 KDA GELATINASE, 92 KDA TYPE IV COLLAGENASE)	2.19	6.79	8.78
SRV_02516_at	HEAT SHOCK 70 KDA PROTEIN 5 (GLUCOSE-REGULATED PROTEIN, 78 KDA)	2.70	10.28	7.60
SRV_04970_a_at	CASPASE RECRUITMENT DOMAIN FAMILY, MEMBER 6	1.35	4.78	4.80
SRV_08154_a_at	HEAT SHOCK 70 KDA PROTEIN 9B (MORTALIN-2)	1.18	3.45	2.71
SRV_04300_a_at	CYTOCHROME C, SOMATIC	1.30	2.80	2.64
SRV_02132_at	EUKARYOTIC TRANSLATION ELONGATION FACTOR 1 EPSILON 1	1.21	3.56	3.38
SRV_01812_a_at	HEAT SHOCK PROTEIN 90 KDA BETA (GRP94), MEMBER 1	1.31	3.53	3.26
SRV_03753_at	GROWTH ARREST AND DNA-DAMAGE-INDUCIBLE, BETA	1.39	7.34	8.89
SRV_03023_a_at	GROWTH ARREST AND DNA-DAMAGE-INDUCIBLE, GAMMA	1.00	3.73	5.99
				
metabolic process				
SRV_05147_a_at	SYNDECAN BINDING PROTEIN (SYNTENIN)	1.27	4.78	4.42
SRV_00332_a_at	glucose-6-phosphate dehydrogenase	1.32	4.55	3.35
SRV_00292_a_at	PHOSPHOMANNOMUTASE 2	1.14	2.61	2.65
SRV_01737_a_at	UDP-N-ACTEYLGLUCOSAMINE PYROPHOSPHORYLASE 1	1.84	2.18	2.59
SRV_05108_a_at	PHOSPHOSERINE AMINOTRANSFERASE 1	1.12	1.97	2.48
C_s_at	ALDOLASE A, FRUCTOSE-BISPHOSPHATE	1.15	2.42	2.27
A_s_at	ALDOLASE A, FRUCTOSE-BISPHOSPHATE	1.24	3.64	3.39
SRV_00129_a_at	ALDOLASE B, FRUCTOSE-BISPHOSPHATE	1.26	2.30	2.13
SRV_02002_at	CARBOXYLESTERASE 1 (MONOCYTE/MACROPHAGE SERINE ESTERASE 1)	1.28	3.86	2.38
				
signal transduction				
SRV_02657_at	PRE-B-CELL COLONY ENHANCING FACTOR 1	1.61	7.61	7.01
SRV_03054_at	MACROPHAGE RECEPTOR WITH COLLAGENOUS STRUCTURE	2.17	8.20	6.82
SRV_01818_at	THIOREDOXIN	1.19	3.29	3.51
SRV_01313_a_at	GRANULIN	1.45	3.72	3.40
SRV_01462_at	NUCLEAR FACTOR OF KAPPA LIGHT POLYPEPTIDE GENE ENHANCER IN B-CELLS 2 (P49/P100)	1.59	3.67	2.77
SRV_03006_a_at	IQ MOTIF CONTAINING GTPASE ACTIVATING PROTEIN 2	1.23	2.59	2.69
SRV_01480_at	PURINERGIC RECEPTOR P2Y, G-PROTEIN COUPLED, 2	1.47	3.17	2.44
SRV_02894_a_at	CAP, ADENYLATE CYCLASE-ASSOCIATED PROTEIN 1 (YEAST)	1.10	2.09	1.76
SRV_00844_a_at	CHLORIDE INTRACELLULAR CHANNEL 1	1.15	1.95	2.10
				
membrane				
SRV_03227_at	NIDOGEN 2 (OSTEONIDOGEN)	1.42	3.69	4.29
SRV_01179_a_at	CD63 ANTIGEN (MELANOMA 1 ANTIGEN)	1.34	3.82	3.97
SRV_02687_s_at	MANNOSE-6-PHOSPHATE RECEPTOR BINDING PROTEIN 1	1.30	4.82	3.55
SRV_04888_a_at	UDP-GLCNAC:BETAGAL BETA-1,3-N-ACETYLGLUCOSAMINYLTRANSFERASE 5	2.41	4.44	3.50
SRV_04819_a_at	TRANSMEMBRANE PROTEIN 49	1.46	3.40	3.20
SRV_04070_a_at	LEUCINE RICH REPEAT CONTAINING 59	1.22	2.48	2.78
SRV_03407_at	FER-1-LIKE 3, MYOFERLIN (C. ELEGANS)	1.22	2.67	3.81
SRV_05439_at	HYPOTHETICAL PROTEIN LOC441168	1.40	3.43	2.79
SRV_02874_a_at	BRAIN ABUNDANT, MEMBRANE ATTACHED SIGNAL PROTEIN 1	1.33	2.80	2.31
SRV_04226_a_at	GTPASE, IMAP FAMILY MEMBER 5	1.13	2.05	2.02
				
transport				
SRV_00744_a_at	ADIPOSE DIFFERENTIATION-RELATED PROTEIN	3.49	12.49	7.34
SRV_00294_s_at	PROTECTIVE PROTEIN FOR BETA-GALACTOSIDASE (GALACTOSIALIDOSIS)	1.53	5.23	4.73
SRV_02592_a_at	LYSYL OXIDASE-LIKE 1	1.18	2.81	4.59
SRV_03991_a_at	SOLUTE CARRIER FAMILY 15, MEMBER 3	1.36	5.33	3.88
SRV_03562_at	ERO1-LIKE (S. CEREVISIAE)	1.00	2.24	1.77
SRV_04996_at	SOLUTE CARRIER FAMILY 7 (CATIONIC AMINO ACID TRANSPORTER, Y+ SYSTEM), MEMBER 3	1.07	1.75	2.11
SRV_01134_at	ATPASE, H+ TRANSPORTING, LYSOSOMAL 56/58 KDA, V1 SUBUNIT B2	1.04	2.17	1.59
SRV_01220_at	CYTOCHROME C OXIDASE SUBUNIT VIB POLYPEPTIDE 1 (UBIQUITOUS)	1.15	4.51	4.18
SRV_02133_a_at	GOLGI SNAP RECEPTOR COMPLEX MEMBER 2	1.05	2.24	1.88
				
extracellular region				
SRV_02948_a_at	fibulin 1	0.93	1.65	2.24
SRV_01275_at	fibulin 1	0.81	1.54	2.10
SRV_02965_at	TISSUE FACTOR PATHWAY INHIBITOR 2	3.56	3.04	2.56
SRV_03142_at	ELASTIN MICROFIBRIL INTERFACER 1	1.11	3.37	3.73
				
ion binding				
SRV_00713_a_at	TRANSCOBALAMIN I (VITAMIN B12 BINDING PROTEIN, R BINDER FAMILY)	2.55	23.02	12.98
SRV_02456_at	GASTRIC INTRINSIC FACTOR (VITAMIN B SYNTHESIS)	3.36	20.67	11.63
SRV_07722_at	CHROMOSOME 17 OPEN READING FRAME 27	0.94	4.46	4.63
SRV_05460_at	tripartite motif-containing 39	1.58	3.81	4.07
SRV_05065_at	tripartite motif-containing 17	1.53	2.86	3.46
SRV_00371_a_at	FRUCTOSE-1,6-BISPHOSPHATASE 1	2.40	5.01	3.14
SRV_00741_a_at	ADENOSINE DEAMINASE, RNA-SPECIFIC	1.44	3.76	2.86
SRV_00562_a_at	PROSTAGLANDIN-ENDOPEROXIDE SYNTHASE 1 (PROSTAGLANDIN G/H SYNTHASE AND CYCLOOXYGENASE)	1.29	2.05	2.54
SRV_05448_at	ATPase, Ca++ transporting, cardiac muscle, slow twitch 2	0.87	2.16	2.13
SRV_02724_at	METHIONINE ADENOSYLTRANSFERASE II, ALPHA	1.66	2.06	1.56
SRV_00449_a_at	LATENT TRANSFORMING GROWTH FACTOR BETA BINDING PROTEIN 1	1.05	1.79	3.15
SRV_00131_a_at	SECRETED PROTEIN, ACIDIC, CYSTEINE-RICH (OSTEONECTIN)	1.19	1.99	2.74
SRV_04896_a_at	TRANSKETOLASE (WERNICKE-KORSAKOFF SYNDROME)	1.44	4.02	2.52
SRV_02047_at	ANNEXIN A2	1.29	2.15	2.45
SRV_01345_at	INOSITOL 1,4,5-TRIPHOSPHATE RECEPTOR, TYPE 3	1.19	2.80	2.35
SRV_12418_at	MYOSIN, LIGHT POLYPEPTIDE 9, REGULATORY	1.01	1.99	2.20
SRV_01649_at	RETICULOCALBIN 1, EF-HAND CALCIUM BINDING DOMAIN	1.16	3.01	2.68
				
Protein binding/transport				
SRV_03206_at	V-SET AND IMMUNOGLOBULIN DOMAIN CONTAINING 4	1.90	13.14	9.17
SRV_00797_a_at	SERPIN PEPTIDASE INHIBITOR, CLADE H (HEAT SHOCK PROTEIN 47), MEMBER 1, (COLLAGEN BINDING PROTEIN 1)	1.77	4.67	4.79
SRV_04964_a_at	TUBULIN, BETA 6	1.06	2.53	2.55
SRV_03477_a_at	TRANSMEMBRANE PROTEIN 4	1.59	2.22	2.39
SRV_02085_at	FK506 BINDING PROTEIN 1B, 12.6 KDA	1.29	1.71	2.18
SRV_02652_a_at	PROTEIN DISULFIDE ISOMERASE FAMILY A, MEMBER 6	1.28	2.16	1.99
SRV_02814_at	DNAJ (HSP40) HOMOLOG, SUBFAMILY B, MEMBER 1	2.35	4.29	1.73
SRV_03255_a_at	AHA1, ACTIVATOR OF HEAT SHOCK 90 KDA PROTEIN ATPASE HOMOLOG 1 (YEAST)	1.07	2.14	1.53
SRV_05534_at	HYPOTHETICAL PROTEIN MGC16212	1.74	4.05	3.73
SRV_00840_a_at	COLD INDUCIBLE RNA BINDING PROTEIN	1.03	2.46	3.20
SRV_01147_a_at	BIGLYCAN	0.99	2.01	2.90
SRV_05461_x_at	tripartite motif-containing 39	1.32	2.53	2.76
SRV_02070_a_at	CYSTEINE AND GLYCINE-RICH PROTEIN 1	1.27	2.22	2.46
SRV_00257_at	KERATIN 18	1.12	1.93	2.44
SRV_04005_at	KDEL (LYS-ASP-GLU-LEU) ENDOPLASMIC RETICULUM PROTEIN RETENTION RECEPTOR 3	0.90	2.07	2.06
SRV_02647_a_at	ARP2 ACTIN-RELATED PROTEIN 2 HOMOLOG (YEAST)	1.23	2.33	1.93
SRV_01234_a_at	CATENIN (CADHERIN-ASSOCIATED PROTEIN), ALPHA 1, 102 KDA	0.98	1.43	2.05
SRV_01504_a_at	PHOSPHOGLUCONATE DEHYDROGENASE	1.19	2.20	1.50
SRV_05174_a_at	THREE PRIME REPAIR EXONUCLEASE 2	1.57	5.55	5.02
				
protein modification				
SRV_04305_a_at	GLYCINE N-METHYLTRANSFERASE	1.24	1.84	3.49
SRV_01832_a_at	UBIQUITIN-CONJUGATING ENZYME E2L 3	1.04	2.09	1.99
SRV_00309_at	TRANSGLUTAMINASE 1 (K POLYPEPTIDE EPIDERMAL TYPE I, PROTEIN-GLUTAMINE-GAMMA-GLUTAMYLTRANSFERASE)	3.22	16.60	14.36
SRV_02093_a_at	HEAT SHOCK 70 KDA PROTEIN 9B (MORTALIN-2)	1.35	3.91	2.79
SRV_02989_at	HEAT SHOCK 70 KDA PROTEIN 8	2.28	6.93	2.73
SRV_01225_at	CRYSTALLIN, ALPHA B	1.27	3.35	2.38
SRV_05456_a_at	UBIQUITIN SPECIFIC PEPTIDASE 2	1.27	2.56	2.00
SRV_05457_a_at	UBIQUITIN SPECIFIC PEPTIDASE 2	1.26	2.28	1.89
SRV_11417_a_at	MATRIX METALLOPEPTIDASE 1 (INTERSTITIAL COLLAGENASE)	5.26	13.96	3.65
SRV_04306_at	GLYCINE N-METHYLTRANSFERASE	1.03	2.15	4.66
SRV_00327_a_at	CATHEPSIN K (PYCNODYSOSTOSIS)	1.37	3.23	3.94
				
cellular process				
SRV_11663_a_at	TIMP METALLOPEPTIDASE INHIBITOR 1	1.96	24.74	23.31
SRV_04387_at	RETINOIC ACID RECEPTOR RESPONDER (TAZAROTENE INDUCED) 1	1.64	6.63	9.22
SRV_03285_at	RELATED RAS VIRAL (R-RAS) ONCOGENE HOMOLOG 2	1.76	5.07	4.97
SRV_04911_at	SIMILAR TO THIOREDOXIN DOMAIN-CONTAINING 2	1.34	3.15	3.20
SRV_01534_at	PROTEIN PHOSPHATASE 1, CATALYTIC SUBUNIT, ALPHA ISOFORM	1.15	2.04	2.18
SRV_04858_at	POLY (ADP-RIBOSE) POLYMERASE FAMILY, MEMBER 9	0.97	2.36	2.15
SRV_03421_a_at	LR8 PROTEIN	1.31	1.95	2.02
SRV_11406_at	V-YES-1 YAMAGUCHI SARCOMA VIRAL RELATED ONCOGENE HOMOLOG	1.37	2.46	2.12
				
other				
SRV_03887_at	DYNEIN, CYTOPLASMIC 1, LIGHT INTERMEDIATE CHAIN 1	1.31	2.42	2.20
SRV_01920_at	poly (ADP-ribose) glycohydrolase	1.31	1.95	2.23
SRV_00155_a_at	COAGULATION FACTOR XIII, A1 POLYPEPTIDE	1.20	2.46	2.35
SRV_01367_a_at	KERATIN 8	1.19	2.90	3.29
SRV_00775_at	ARGINASE, TYPE II	1.44	4.08	6.06
SRV_11767_a_at	INTERFERON INDUCED TRANSMEMBRANE PROTEIN 5	1.17	2.74	4.24
SRV_07726_a_at	MACROPHAGE EXPRESSED GENE 1	1.69	4.25	3.18
SRV_01302_at	GUANYLATE BINDING PROTEIN 4	1.07	2.39	2.64
SRV_02761_at	ARGININE-RICH, MUTATED IN EARLY STAGE TUMORS	1.10	2.17	1.99
SRV_03758_a_at	ARRESTIN DOMAIN CONTAINING 2	0.90	5.48	8.72

**Table 2:** Genes which were significantly down-regulated at any time point. Numbers indicate fold change at that time point.

ID	NAME	24 hr	72 hr	144 hr
transcription				
SRV_04230_at	CHROMOSOME X OPEN READING FRAME 15	0.73	0.44	0.50
SRV_01344_a_at	INTERFERON REGULATORY FACTOR 2	1.02	0.47	0.50
SRV_01768_a_at	TAF9 RNA POLYMERASE II, TATA BOX BINDING PROTEIN (TBP)-ASSOCIATED FACTOR, 32 KDA	0.84	0.44	0.50
SRV_03843_a_at	MEDIATOR OF RNA POLYMERASE II TRANSCRIPTION, SUBUNIT 31 HOMOLOG (YEAST)	0.95	0.50	0.45
SRV_01892_at	ZINC FINGER PROTEIN 282	0.98	0.39	0.42
				
translation				
SRV_03800_a_at	MITOCHONDRIAL RIBOSOMAL PROTEIN S7	1.13	0.50	0.54
SRV_03598_at	MITOCHONDRIAL RIBOSOMAL PROTEIN L19	0.73	0.49	0.53
SRV_04607_at	PEPTIDE DEFORMYLASE-LIKE PROTEIN	1.01	0.46	0.50
SRV_04925_a_at	HYPOTHETICAL PROTEIN MGC11102	1.01	0.43	0.48
SRV_01958_at	EUKARYOTIC TRANSLATION INITIATION FACTOR 4E BINDING PROTEIN 3	1.05	0.32	0.31
				
Natural Killer cell mediated cytotoxicity				
AE_at	TUBULIN, BETA 2C	0.88	0.52	0.49
				
Apoptosis				
SRV_11815_at	CASP2 AND RIPK1 DOMAIN CONTAINING ADAPTOR WITH DEATH DOMAIN	0.70	0.45	0.52
SRV_01489_at	PRKC, APOPTOSIS, WT1, REGULATOR	0.88	0.43	0.44
				
ion binding/transport				
SRV_03020_at	TRAF-TYPE ZINC FINGER DOMAIN CONTAINING 1	0.85	0.49	0.53
SRV_01742_at	SPECTRIN, ALPHA, NON-ERYTHROCYTIC 1 (ALPHA-FODRIN)	0.82	0.46	0.52
SRV_02131_a_at	PEPTIDASE (MITOCHONDRIAL PROCESSING) BETA	0.80	0.48	0.51
SRV_02733_at	MITOCHONDRIAL INTERMEDIATE PEPTIDASE	0.59	0.39	0.40
SRV_04112_at	HYPOTHETICAL PROTEIN FLJ20699	0.93	0.42	0.39
SRV_00559_a_at	PRIMASE, POLYPEPTIDE 1, 49 KDA	0.79	0.42	0.39
SRV_03126_at	RING FINGER PROTEIN 113A	0.95	0.39	0.39
SRV_03759_at	ATP SYNTHASE, H+ TRANSPORTING, MITOCHONDRIAL F0 COMPLEX, SUBUNIT S (FACTOR B)	0.91	0.30	0.25
SRV_01177_a_at	ECTONUCLEOSIDE TRIPHOSPHATE DIPHOSPHOHYDROLASE 1	1.19	0.52	0.49
SRV_04638_a_at	MEMBRANE-ASSOCIATED RING FINGER (C3HC4) 7	0.78	0.44	0.47
SRV_03403_at	MAKORIN, RING FINGER PROTEIN, 1	1.02	0.55	0.48
SRV_02137_at	GUANINE DEAMINASE	0.93	0.36	0.36
SRV_12156_at	TUMOR PROTEIN D52	0.93	0.58	0.45
SRV_02173_at	CHONDROITIN SULFATE PROTEOGLYCAN 3 (NEUROCAN)	1.38	0.57	0.20
				
metabolic process				
SRV_02346_a_at	GLUTATHIONE S-TRANSFERASE OMEGA 1	1.04	0.54	0.47
SRV_04215_at	PHOSPHOGLUCOMUTASE 2	1.08	0.57	0.46
SRV_04397_at	NITRILASE FAMILY, MEMBER 2	0.86	0.49	0.46
SRV_03369_at	NON-METASTATIC CELLS 7, PROTEIN EXPRESSED IN (NUCLEOSIDE-DIPHOSPHATE KINASE)	1.00	0.55	0.43
SRV_00123_at	AMINOLEVULINATE, DELTA-, DEHYDRATASE	0.71	0.49	0.31
SRV_00160_s_at	FUMARYLACETOACETATE HYDROLASE (FUMARYLACETOACETASE)	0.83	0.61	0.49
SRV_00135_at	ASPARTOACYLASE (CANAVAN DISEASE)	0.92	0.30	0.22
SRV_01499_at	6-PHOSPHOFRUCTO-2-KINASE/FRUCTOSE-2,6-BIPHOSPHATASE 1	0.88	0.65	0.47
SRV_11745_at	ACYL-COENZYME A OXIDASE 3, PRISTANOYL	0.87	0.51	0.47
SRV_03094_at	LIPOIC ACID SYNTHETASE	0.83	0.47	0.47
SRV_05217_a_at	SERINE DEHYDRATASE-LIKE	1.03	0.52	0.33
				
transport				
SRV_03906_at	HEMATOPOIETIC STEM/PROGENITOR CELLS 176	0.81	0.49	0.58
SRV_04743_a_at	HYPOTHETICAL PROTEIN FLJ22028	0.98	0.54	0.50
SRV_02065_a_at	ADAPTOR-RELATED PROTEIN COMPLEX 2, MU 1 SUBUNIT	0.90	0.59	0.48
SRV_03218_a_at	GABA(A) RECEPTOR-ASSOCIATED PROTEIN-LIKE 2	0.93	0.28	0.37
SRV_05300_a_at	SFT2 DOMAIN CONTAINING 2	0.91	0.31	0.29
SRV_05537_a_at	TRAFFICKING PROTEIN PARTICLE COMPLEX 6B	0.95	0.49	0.34
SRV_02033_a_at	SELENIUM BINDING PROTEIN 1	1.05	0.58	0.45
				
protein binding/modification				
SRV_04235_at	hypothetical protein FLJ11280	0.68	0.44	0.56
SRV_02678_a_at	M-PHASE PHOSPHOPROTEIN 6	0.84	0.45	0.51
SRV_02198_a_at	GLUTAMYL-PROLYL-TRNA SYNTHETASE	0.94	0.52	0.48
SRV_01481_at	PHOSPHATIDYLETHANOLAMINE BINDING PROTEIN 1	1.05	0.56	0.49
SRV_01495_at	PYRUVATE DEHYDROGENASE KINASE, ISOZYME 2	1.04	0.41	0.31
SRV_04077_a_at	UBIQUITIN-CONJUGATING ENZYME E2R 2	0.78	0.51	0.47
SRV_04977_s_at	CNDP DIPEPTIDASE 2 (METALLOPEPTIDASE M20 FAMILY)	1.27	0.49	0.22
SRV_04977_at	CNDP DIPEPTIDASE 2 (METALLOPEPTIDASE M20 FAMILY)	1.20	0.46	0.22
SRV_01825_at	UBIQUITIN-CONJUGATING ENZYME E2B (RAD6 HOMOLOG)	0.90	0.47	0.51
				
RNA binding/processing				
SRV_03823_at	RNA BINDING MOTIF PROTEIN, X-LINKED 2	0.80	0.47	0.54
SRV_03721_at	SYF2 HOMOLOG, RNA SPLICING FACTOR (S. CEREVISIAE)	0.96	0.46	0.49
SRV_03417_at	MITOCHONDRIAL RIBOSOMAL PROTEIN S28	0.86	0.51	0.47
SRV_03836_at	EXOSOME COMPONENT 1	1.03	0.46	0.46
				
cell cycle/cell division				
SRV_05218_a_at	COILED-COIL DOMAIN CONTAINING 5 (SPINDLE ASSOCIATED)	0.68	0.36	0.46
SRV_03244_a_at	FREQUENTLY REARRANGED IN ADVANCED T-CELL LYMPHOMAS 2	0.89	0.38	0.46
SRV_05024_at	ZW10 INTERACTOR	0.96	0.41	0.44
SRV_00804_at	CDC6 CELL DIVISION CYCLE 6 HOMOLOG (S. CEREVISIAE)	0.70	0.50	0.42
SRV_03256_at	TPX2, MICROTUBULE-ASSOCIATED, HOMOLOG (XENOPUS LAEVIS)	0.91	0.41	0.51
SRV_04156_at	CELL DIVISION CYCLE ASSOCIATED 8	0.71	0.43	0.50
SRV_03593_at	DISCS, LARGE HOMOLOG 7 (DROSOPHILA)	0.66	0.39	0.49
SRV_14350_at	NIMA (NEVER IN MITOSIS GENE A)-RELATED KINASE 3	0.96	0.42	0.30
SRV_02556_at	SMC4 STRUCTURAL MAINTENANCE OF CHROMOSOMES 4-LIKE 1 (YEAST)	0.69	0.43	0.47
SRV_03257_at	TPX2, MICROTUBULE-ASSOCIATED, HOMOLOG (XENOPUS LAEVIS)	0.79	0.35	0.45
SRV_02235_at	KINESIN FAMILY MEMBER 11	0.53	0.31	0.44
SRV_01290_at	FERRITIN, HEAVY POLYPEPTIDE 1	1.00	0.54	0.42
SRV_02151_a_at	CENTRIN, EF-HAND PROTEIN, 2	0.65	0.39	0.39
SRV_04253_a_at	NUCLEOLAR AND SPINDLE ASSOCIATED PROTEIN 1	0.62	0.28	0.37
SRV_05141_at	CYCLIN-DEPENDENT KINASE INHIBITOR 2C (P18, INHIBITS CDK4)	0.85	0.57	0.37
SRV_00033_copy4_at	T-cell acute lymphocytic leukemia 1	1.02	0.47	0.36
SRV_00033_at	T-cell acute lymphocytic leukemia 1	0.97	0.44	0.35
SRV_00033_copy2_at	T-cell acute lymphocytic leukemia 1	0.96	0.46	0.34
SRV_00033_copy1_at	T-cell acute lymphocytic leukemia 1	0.96	0.44	0.33
SRV_00033_copy3_at	T-cell acute lymphocytic leukemia 1	0.94	0.44	0.33
				
membrane				
SRV_04260_at	CHROMOSOME 9 OPEN READING FRAME 46	1.05	0.56	0.47
SRV_04763_at	CHROMOSOME 10 OPEN READING FRAME 57	1.24	0.46	0.42
SRV_04650_a_at	TRANSMEMBRANE 6 SUPERFAMILY MEMBER 1	1.00	0.47	0.32
SRV_05571_a_at	OXIDATION RESISTANCE 1	0.77	0.37	0.42
SRV_03611_a_at	TRANSLOCASE OF OUTER MITOCHONDRIAL MEMBRANE 70 HOMOLOG A (YEAST)	0.97	0.55	0.48
				
kinase activity				
SRV_05333_at	RIO kinase 3 (yeast)	1.06	0.47	0.52
SRV_05450_a_at	INTEGRIN BETA 1 BINDING PROTEIN 3	0.87	0.53	0.46
SRV_01863_at	VACCINIA RELATED KINASE 1	0.61	0.39	0.51
				
pinocytosis/endocytosis				
SRV_00866_at	DISABLED HOMOLOG 2, MITOGEN-RESPONSIVE PHOSPHOPROTEIN (DROSOPHILA)	0.84	0.49	0.43
				
DNA damage				
SRV_04199_at	NEI ENDONUCLEASE VIII-LIKE 3 (E. COLI)	0.75	0.41	0.37
SRV_02222_at	HUS1 CHECKPOINT HOMOLOG (S. POMBE)	0.72	0.45	0.57
				
muscle development/contraction				
SRV_01033_a_at	INTERFERON-RELATED DEVELOPMENTAL REGULATOR 1	1.18	0.38	0.53
SRV_05143_a_at	MYOSIN, LIGHT POLYPEPTIDE 1, ALKALI; SKELETAL, FAST	0.79	0.50	0.49
SRV_00058_s_at	tropomyosin	0.89	0.53	0.48
				
other				
SRV_01932_a_at	FICOLIN (COLLAGEN/FIBRINOGEN DOMAIN CONTAINING) 3 (HAKATA ANTIGEN)	1.03	0.58	0.46
SRV_05356_s_at	FAMILY WITH SEQUENCE SIMILARITY 58, MEMBER A	0.89	0.46	0.47
SRV_02972_at	GLUTAREDOXIN 5 HOMOLOG (S. CEREVISIAE)	0.97	0.49	0.49
SRV_05356_at	FAMILY WITH SEQUENCE SIMILARITY 58, MEMBER A	1.09	0.58	0.58
SRV_05263_at	SOLUTE CARRIER FAMILY 39 (ZINC TRANSPORTER), MEMBER 3	0.73	0.45	0.51
SRV_04739_a_at	ZINC FINGER PROTEIN 403	0.97	0.49	0.51
SRV_03830_at	SHWACHMAN-BODIAN-DIAMOND SYNDROME	0.94	0.47	0.46
SRV_03448_at	COILED-COIL DOMAIN CONTAINING 59	0.89	0.42	0.44
SRV_02223_a_at	ISOPENTENYL-DIPHOSPHATE DELTA ISOMERASE 1	0.89	0.37	0.43
SRV_04160_at	SDA1 DOMAIN CONTAINING 1	0.88	0.48	0.42
SRV_05216_at	SERUM AMYLOID A-LIKE 1	0.82	0.42	0.42
SRV_04991_a_at	MYC INDUCED NUCLEAR ANTIGEN	0.90	0.41	0.39
SRV_00134_a_at	ARGININOSUCCINATE LYASE	1.10	0.44	0.36
SRV_05376_at	WILLIAMS BEUREN SYNDROME CHROMOSOME REGION 27	0.84	0.36	0.31

**Table 3:** Significant (p ≤ 0.05 geometric mean p-value) functional groups obtained from functional annotation using DAVID.

	Ontology	Number of Genes	p-value		Ontology	Number of Genes	p-value
Functional Group 1	response to biotic stimulus	25	<0.001	Functional Group 5	water-soluble vitamin metabolism	4	0.02
<0.001	immune response	21	<0.001	0.033	vitamin metabolism	4	0.03
	defense response	22	<0.001		pyridine nucleotide metabolism	3	0.07
							
Functional Group 2	cation binding	34	0.001	Functional Group 6	di-, tri-valent inorganic cation transport	5	0.006
0.002	ion binding	36	0.004	0.042	metal ion transport	5	0.06
	metal ion binding	36	0.004		cation transport	6	0.22
							
Functional Group 3	innate immunity	4	0.001	Functional Group 7	bcr protein	3	0.02
0.005	immune response	5	0.004	0.045	molecular chaperone	4	0.02
	innate immune response	4	0.007		Heat shock protein Hsp70	3	0.03
	complement activation	3	0.02		Heat shock protein 70	3	0.03
					antigen processing and presentation	4	0.09
					cell surface	3	0.20
							
Functional Group 4	lysosome	7	0.002				
0.008	lysosome	7	0.009	Functional Group 8	response to unfolded protein	5	0.02
	lytic vacuole	7	0.009	0.048	response to protein stimulus	5	0.02
	vacuole	7	0.02		chaperone	6	0.26

### Genes Up-regulated in Response to ATV

Across all time points, the majority of up-regulated genes were related to immune response or other related functions, such as inflammation and apoptosis. Other up-regulated genes pertained to gene functions such as ion binding and transport, membrane related functions, and protein binding and modification. Twenty-three genes (represented by 26 probe sets) demonstrated 2-fold or greater changes at 24 hours post-infection, all of which were up-regulated. Ten of these 23 have functions pertaining to immune response. Of the remaining highly expressed genes, one was associated with inflammation, two to regulation of apoptosis, three to ion binding, three to protein binding and modification, one to transport, one to the extracellular constituent, and one to membrane and glycolipids. Many of these genes showed increasing transcript abundances over time. At 72 hours post infection, 43 genes had a greater than 5-fold change, and 40 genes had a greater than 5-fold change at 144 hours. The highest expression level, 91-fold increase at 144 hours, was observed for *interferon-induced protein with tetracopeptide repeats 5 *(IFIT5).

### Genes Down-regulated in Response to ATV

In contrast to the very high fold changes observed among up-regulated genes, the largest fold change observed among down-regulated genes was approximately 4.9-fold, in *chondroitin sulfate proteoglycan *(NCAN). Five down-regulated genes each code for regulation of transcription and translation. An additional 15 down-regulated genes correspond to 20 probe sets that have functions associated with cell division and mitosis, which was not observed in the up-regulated genes. Other notable down-regulated gene ontologies include one gene corresponding to pinocytosis and endocytosis, and one gene related to natural killer cell mediated cytotoxicity.

### Validation of Microarray Results Using Quantitative Real-time PCR

We used qPCR to estimate fold changes for nine genes to verify our microarray data (Table 4). For five of the nine genes investigated (56%; *Myxovirus resistance 1, Macrophage receptor with collagenous structure, Complement component 3, Cyclin dependant kinase inhibitor 1B, Vaccinia related kinase 1*) there is good agreement between the microarray and qPCR data. In genes where the microarray estimates of fold change were modest (*Serine dehydratase like, Hemoglobin gamma alpha, Glycogen synthase kinase, Programmed cell death 8*) there is poorer agreement between fold change estimates from these two technologies. However, for this latter group of genes with modest fold change values, the microarray and qPCR data were always within four fold of each other. These results demonstrate that we were able to verify robust differences that were suggested by the microarray data.

**Table 4:** Fold changes obtained from microarray and from quantitative real-time PCR.

Gene name		Microarray	qPCR	
	24	72	24	72
	
Myxovirus resistance 1 MX1	3.13	29.23	5.97	24.44
Macrophage receptor with collagenous structure MARCO	2.17	8.20	3.36	15.24
Complement component 3 C3	1.80	9.79	2.14	14.78
Cyclin dependant kinase inhibitor 1B CDKN1B	-1.17	-1.74	-2.26	-3.16
Vaccinia related kinase 1 VRK1	-1.64	-2.58	-1.23	-1.88
Serine dehydratase like SDSL	1.03	-1.92	-1.35	-1.01
Hemoglobin gamma alpha HBG1	-1.01	-1.02	-1.54	-1.87
Glycogen synthase kinase GSK3A	-1.07	-1.08	-1.13	2.76
Programmed cell death 8 PDCD8	1.13	-1.10	1.30	1.85

### Analyses to identify proliferation gene expression signatures

Comparison of gene expression after ATV and tail amputation identified 25 genes that are significantly up-regulated in both experimental frameworks (Table 5). No significantly down-regulated genes were identified in common. Several of the commonly up-regulated genes appear to be related to humoral immunity, and membrane and extracellular matrix related functions. Additionally, general stress response genes such as *heat shock 70 kDa protein 5 *were similarly regulated. None of the cell cycle genes that are significantly up-regulated during tail regeneration were identified in this study. Thus, there was no evidence of cell proliferation by spleen cells after ATV infection.

**Table 5:** Genes expressed in both ATV infection and spinal cord injury

Gene ID	Gene Name	Gene Ontology
SRV_00294_s_at	protective protein for beta-galactosidase (galactosialidosis)	proteolysis, protein transport
SRV_00309_at	transglutaminase 1 (K polypeptide epidermal type I, protein-glutamine-gamma-glutamyltransferase)	membrane, cell envelope, protein modification
SRV_00327_a_at	cathepsin K (pycnodysostosis)	proteolysis
SRV_00330_at	cytochrome b-245, beta polypeptide (chronic granulomatous disease)	humoral response, inflammatory response
SRV_00371_a_at	fructose-1,6-bisphosphatase 1	metal ion binding (zinc)
SRV_00442_at	solute carrier family 11 (proton-coupled divalent metal ion transporters), member 1	immune response, ion transport
SRV_00713_a_at	transcobalamin I (vitamin B12 binding protein, R binder family)	ion transport/binding (cobalt)
SRV_00744_a_at	adipose differentiation-related protein	fatty acid transport, extracellular region
SRV_01179_a_at	CD63 antigen (melanoma 1 antigen)	endosome, membrane
SRV_01342_at	interferon regulatory factor 1	immune response, transcription
SRV_01351_at	jun B proto-oncogene	transcription
SRV_01818_at	thioredoxin	signal transduction
SRV_02399_a_at	matrix metalloproteinase 9 (gelatinase B, 92 kDa gelatinase, 92 kDa type IV collagenase)	extracellular matrix, apoptosis, proteolysis
SRV_02456_at	gastric intrinsic factor (vitamin B synthesis)	ion transport/binding (cobalt)
SRV_02516_at	heat shock 70 kDa protein 5 (glucose-regulated protein, 78 kDa)	anti-apoptosis, endoplasmic reticulum
SRV_02586_at	lectin, galactoside-binding, soluble, 3 binding protein	cell adhesion, cellular defense response, signal transduction
SRV_03054_at	macrophage receptor with collagenous structure	signal transduction
SRV_04604_at	interferon induced with helicase c domain 1	innate immune response, regulation of apoptosis, response to virus
SRV_04819_a_at	transmembrane protein 49	membrane, endoplasmic reticulum
SRV_04888_a_at	UDP-GlcNAc:betaGal beta-1,3-N-acetylglucosaminyltransferase 5	membrane, CNS development
SRV_04911_at	thioredoxin domain containing 2 (spermatozoa)	cell redox homeostasis
SRV_04964_a_at	tubulin beta MGC4083	nucleotide binding, protein polymerization
SRV_07726_a_at	macrophage expressed gene 1	none
SRV_11417_a_at	matrix metalloproteinase 1 (interstitial collagenase)	proteolysis, ion binding (zinc)
SRV_11663_a_at	tissue inhibitor of metalloproteinase 1 (erythroid potentiating activity, collagenase inhibitor)	enzyme inhibitor, cell proliferation
SRV_00294_s_at	protective protein for beta-galactosidase (galactosialidosis)	proteolysis, protein transport

## Discussion and conclusion

Emerging infectious diseases are implicated in the global decline of amphibians and other animals [3,49-51]. There is urgent need to develop understanding of amphibian immunological responses to pathogens and to identify host genes that may be important in disease resistance. Our study shows that functional genomics provides a means to rapidly meet these needs. We infected Mexican axolotls from the Ambystoma Genetic Stock Center with a viral pathogen that is clearly affecting tiger salamander populations in nature [10,13-15,19]. Our results show that ATV infection induces transcriptional changes of genes that are known to function in vertebrate immunity. Below we discuss the transcriptional response in more detail and suggest hypotheses to explain why ATV is often lethal to axolotls and other tiger salamanders.

We detected significant gene expression changes 24 hours post infection. Many of these gene expression changes likely reflect transcription within lymphocytes, as they are the predominant cell type in the spleen of juvenile and adult axolotls [52]. Indeed, the functions of many of these genes are associated with neutrophil, dendritic, and macrophage cell functions, including cytokine signaling (*chemokine (C-X-C motif) receptor 4*), phagocytosis and destruction of phagocytised particles (*disabled homolog 2, mitogen-responsive phosphoprotein, neutrophil cytosolic factor 2*, *lysosomal-associated membrane protein 1*, *RAS homolog gene family, member B*), complement (*complement factor B*, *complement component 3*), and inflammation (*pentraxin related gene, rapidly induced by IL-1 beta*, *cytochrome B-245 beta polypeptide*, *n-myc and STAT interactor*). Up-regulation of complement components that are known to function in the removal of viral particles, and up-regulation of the stress-associated transcription factor *jun-b*, clearly shows that ATV induced a humoral gene expression response in the axolotl. Further support for this idea was obtained by comparing ATV-induced gene expression changes to changes identified from a previous microarray experiment using *A. mexicanum *and the same microarray platform. Twenty-five genes that were up-regulated in response to ATV infection were also identified as significantly up-regulated during regeneration [31]. In both microarray studies, blood was not perfused from tissues prior to tissue collection and it is known that leukocyctes express genes during the early wound-healing phase of spinal cord and limb regeneration. Thus, it seems likely that many of the early gene expression changes that we observed in response to ATV-infection reflect a general, humoral transcriptional response to stress.

In addition to this general humoral response, **t**he gene expression patterns that we observed suggest that the Mexican axolotl manifests an antiviral transcriptional response that is not unlike that observed in other vertebrates. For example, ATV infection clearly induces an interferon-mediated, antiviral response. Although probe sets for interferon genes are not represented on the GeneChip, we estimate based upon literature surveys that at least 20% of the significant genes that we identified are known in other systems (*in vitro *and *in vivo*) to be involved in interferon-mediated transcription [53-55]. These genes exhibited some of the largest fold-changes and include two primary transcription factors that compete to activate (*interferon regulatory factor 1*, up-regulated) and repress (*interferon regulatory factor 2*, down-regulated) transcription of interferon-alpha and beta (Type 1 interferon), and inferon-inducible genes that recognize and degrade intra-cellular viral nucleic acid (*interferon induced with helicase C domain 1*). Considering further that four of the most highly enriched functional groups also contained genes relating to the immune response and pathogen response, the results show that axolotls mount a robust anti-viral response from 24–144 hours post-infection.

Given the robust immunological transcription response that we observed, it is curious why ATV is so virulent to tiger salamanders. In the closely related *Ranavirus *frog virus 3 (FV3), larval *Xenopus laevis *succomb to FV3 but adults effectively clear virons and develop lasting resistance to future infection [56]. Adult resistance in *X. laevis *is correlated with a significant proliferation of cytotoxic CD8^+ ^T cells in the spleen upon infection (within 6 days), as well as increased mortality upon CD8^+ ^T cell depletion [30,57]. Mortality events due to ATV are more significant among larvae in natural tiger salamander populations, however metamorphosed adult tiger salamanders are more susceptible than larvae to ATV infection in the lab [18]. It is well established that Mexican axolotls have a less complicated immune system and never develop the type of mature immune response typical of amniote vertebrates [21-29,52]. We did not observe any gene expression changes that would indicate proliferative leukocyte responses in axolotl spleen. Perhaps this is because we used juvenile axolotls that are incapable of such a response. However, it is also possible that ATV maybe more resistant to the immune response mounted by *A. mexicanum *than FV3 is to the *Xenopus *immune response. Phylogenetic analyses indicate ATV is more closely related to iridoviruses found in fish than to FV3, which suggests a relatively recent host switch occurring with the introduction of sportfish to areas of the southwestern United States [15]. Iridoviruses found in sportfish have a larger genome and contain more ORFs related to immune evasion than FV3, which could also be related to improved performance of this virus on the salamander host [15]. Further studies are needed to better understand the ontogeny of immunological responses in axolotls, the virulence of different ranaviruses, and the role of innate versus adaptive immunity in ATV infection.

Our study has identified hundreds of new candidate genes for laboratory and field studies of stress and disease in tiger salamanders. Significantly more gene candidates will undoubtedly be discovered using a higher content, 2^nd ^generation microarray that is currently under development. Genomic and bioinformatics tools make *Ambystoma *a powerful system for wildlife disease research. In particular, molecular information can be quickly cross-referenced from a genetically homogeneous strain that is available for laboratory studies (Mexican axolotl), to other closely related tiger salamander species in North America [20]. Such power is needed to quickly understand how ATV and other pathogens are overwhelming amphibian immune responses and causing population declines in nature.

## Competing interests

The authors declare that they have no competing interests.

## Authors' contributions

JDC performed infections and surgeries and drafted the manuscript. RBP performed microarray statistical and bioinformatic analyses. AS, CKB, and SRV contributed to experimental design and manuscript editing.

## Supplementary Material

Additional file 1 Appendix A. Primer sequences used for qPCR verification of microarray data. Click here for file
