# Supplementary material for: Transcriptional response of Mexican axolotls to Ambystoma tigrinum virus (ATV) infection
Source: BMC Genomics. 2008 Oct 20;9:493. doi: 10.1186/1471-2164-9-493 (PMC2584114; doi:10.1186/1471-2164-9-493)
Supplement: Additional file 1 — Appendix A. Primer sequences used for qPCR verification of microarray data. [file 1471-2164-9-493-S1.doc]

**Appendix A**. Primer sequences used for qPCR verification of microarray data.

| Gene | Primers |  |
| --- | --- | --- |
|  | Foward | Reverse |
| Myxovirus resistance 1 | CATCATCGTGGGCGAGAACT | GGGAAATGCGCTGAAGCA |
| Macrophage receptor with collagenous structure | TCATGACGGGCACGTTGTT | TTTCGGACTCGGACAGTCAAA |
| Complement component 3 | CAACCCAGGTGGTGTTCCAG | CCCGGGCAGTTTTATGGTAAC |
| Cyclin dependant kinase | AGTACAGGCGTTCGGTGCAC | AGTTCCACCGCTGACAGTCC |
| Vaccinia related kinase | ATCGGTATTGTCCTGAAAGAGGAC | ACGTAGGAGCTACGCCCTTG |
| Serine dehydratase | GTGTGCTCGTCAGGTGGAAAT | GAAGCAGACACGCCAAGCTT |
| Hemoglobin gamma alpha | CAAGGTCGCCGCTCATG | TCATCTATGTGGGTGACTGCACTT |
| Glycogen synthase kinase | TGTGTGTTGGCAGAGCTGTTACTT | CTAATTGATCAACACCGCTGTCA |
| Programmed cell death 8 | CAGGATGGCAGAGACGGATC | CCAGATGTTGGAGCGTGCTT |
| Ribosomal protein L19 | CATGGGCACTGGTAAGAGAAAAG | GCGGCGCAAGATTCTCAT |
